# Neonatal Sepsis Caused by *Streptococcus gallolyticus* Complicated with Pulmonary Hypertension: A Case-Report and a Systematic Literature Review

**DOI:** 10.3390/diagnostics12123116

**Published:** 2022-12-10

**Authors:** Zoi Iliodromiti, Marina Tsaousi, Konstantina Kitsou, Helen Bouza, Theodora Boutsikou, Abraham Pouliakis, Efstathia Tsampou, Stavroula Oikonomidi, Maria Dagre, Rozeta Sokou, Nicoletta Iacovidou, Chrysa Petropoulou

**Affiliations:** 1 Neonatal Department, Aretaieio Hospital, National and Kapodistrian University of Athens, 11528 Athens, Greece; 2 Neonatal Intensive Care Unit, “Aghia Sophia” Children’s Hospital, 11527 Athens, Greece; 3 Second Department of Pathology, “Attikon” University Hospital, National and Kapodistrian University of Athens, 12461 Athens, Greece; 4 Department of Biopathology, Aretaieion University Hospital, Medical School, National and Kapodistrian University of Athens, 76, Vasilisis Sofias Avenue, 11528 Athens, Greece

**Keywords:** *Streprococcus gallolyticus*, neonatal sepsis

## Abstract

*Streptococcus gallolyticus* (*S. gallolyticus*) has been linked to the development of infections in adults; however, in neonates *S. gallolyticus* sepsis is very rare and resembles Group B Streptococcal infections. In this case report, we present the case of a full-term neonate who developed early-onset sepsis due to *S. gallolyticus*. A systematic review of the literature was also conducted. The neonate had good APGAR scores at 1′ and 5′. At 5 h postnatally, the neonate developed poor feeding and respiratory distress. She received oxygen in a head box, and a complete blood count and biochemistry, blood, CSF and body surface cultures were obtained. Empiric intravenous antibiotics (ampicillin and tobramycin) were initiated, and she was transferred to a tertiary NICU for further treatment. The neonate was mechanically ventilated and received dopamine and colloid fluids for circulatory support. A cardiology consultation revealed pulmonary hypertension on day one. *S. gallolyticus* was isolated in the blood culture. Central nervous system ultrasonography, brainstem auditory evoked potentials, and a second cardiology evaluation were normal on day three. Clinical and laboratory improvement was noted on day three, and the baby was discharged after a 12-day hospitalization. Follow-up visits were scheduled for reevaluation.

## 1. Introduction

*Streptococcus gallolyticus* (*S. gallolyticus*) is a gram-positive coccus, which belongs to group D Streptococci, frequently found as part of the human intestinal flora, previously known as *Streptococcus bovis* (*S. bovis*) [1]. *S. gallolyticus* has been linked with infective endocarditis and meningitis in adults. Colon cancer is recognized as a risk factor for the development of bacteriemia, resulting in the aforementioned infections [2]. However, *S. gallolyticus* is a rare cause of neonatal infection, both in full- and pre-term neonates, and its clinical presentation resembles that of group B Streptococcal (GBS) infections [1]. We present the case of a full-term female neonate who developed early-onset sepsis due to *S. gallolyticus,* complicated with pulmonary hypertension. We also report all cases of neonatal infection caused by this infectious organism found in the literature.

## 2. Case Presentation

A female neonate was born at 38^+1^ weeks of gestation via cesarian section (CS) due to failure to progress by a primiparous 32-year-old mother with good antenatal follow-up. Rupture of membranes occurred 10 h prior to the CS. Pregnancy history was significant for intrahepatic cholestasis of pregnancy, gestational diabetes mellitus treated with diet, and vaginal bleeding at 30 weeks, which was treated conservatively. Vaginal swab culture at 35^+3^ weeks was negative for pathogenic bacteria.

The neonate cried immediately at birth, and APGAR scores were 9 and 10, at 1′ and 5′, respectively. Birth weight was 3.250 g (65th centile for gestational age, maternal age and somatometric measurements). Skin-to-skin with the mother was performed, the neonate’s physical examination was normal, and the neonate was kept in the mother’s room, as rooming-in is a standard practice in our institution. At the age of 5 h, the neonate developed grunting and poor feeding and was transferred to the neonatal unit of our department for clinical evaluation. Clinical signs of respiratory distress, including tachypnoea (respiratory rate, RR: 75/min) and grunting, were present; heart rate (HR), blood pressure (BP), capillary refilling time (CRT) and temperature were 220/min, 83/41 mmHg (Mean arterial pressure 53 mmHg), 3 s and 39 °C respectively. Oxygen was administered in a head box (FiO_2_ 35%), and complete blood count (CBC), blood biochemistry, and blood, cerebrospinal fluid (CSF), as well as body surface cultures, were obtained. Initial laboratory results were significant for neutropenia (neutrophils 1469/μL). C-Reactive Protein was not elevated (CRP = 0.6 mg/L), and CSF values were within normal limits. Empiric intravenous antibiotics were administered (ampicillin and tobramycin), and the neonate was transferred to a tertiary neonatal intensive care unit (NICU) for further treatment.

On admission to the NICU, the neonate was febrile (38.3 °C), in respiratory distress, with a grade I-II/VI heart murmur, RR 85/min, SpO_2_ 100%, HR 190/min, BP 78/33 (49) mmHg and CRT 4 s. Within a few hours, the neonate’s clinical condition deteriorated rapidly, requiring mechanical ventilation. Sepsis work-up was performed again, and the antibiotic treatment was changed to ampicillin, teicoplanin and cefotaxime for broader coverage. Inotropes (dopamine) and colloid fluids for circulatory support were also initiated. Consultation by a pediatric cardiology specialist revealed patent ductus arteriosus (PDA) of 3 mm, with the left-to-right flow, increased pulmonary mean artery pressure (50 mmHg), normal left ventricular contractility (Ejection Fraction—EF > 65%) while on dopamine treatment, and a small atrial septal defect (ASD).

On the second day of life (DOL), CRP reached peak value (CRP: 41.3 mg/L), and with the application of biochemical testing (Vitek 2), *S. gallolyticus* was isolated in the initial blood culture, sensitive to benzylpenicillin, ampicillin, cefotaxime, levofloxacin, linezolid, teicoplanin, vancomycin, and resistant to tetracycline and clindamycin; further, identification was not performed in the laboratory. According to the antimicrobial susceptibility testing, the neonate’s treatment was changed to ampicillin and cefotaxime. A second cardiology evaluation revealed that ductus arteriosus was constricted, mean pulmonary artery pressure returned to normal, and inotropes support was no longer required. Central nervous system ultrasonography and brainstem auditory evoked potentials study were normal.

Clinical and laboratory improvement was noted from the third DOL. The baby was extubated on the fifth DOL and remained in oxygen for two more days before she was completely weaned from it. Intravenous antibiotic treatment stopped on the tenth DOL. The baby was discharged after a 12-day hospitalization. Follow-up cardiological study was scheduled at three to four months of age, and brainstem auditory evoked potentials study for reevaluation at three months of age; both were performed at the age of three months and were completely normal. Therefore, the infant was discharged without any sequelae.

## 3. Literature Review

### 3.1. Materials and Methods

Our systematic literature review was conducted until 27 October 2022 and followed the protocol proposed by the Preferred Reporting Items for Systematic Reviews and Meta-Analyses (PRISMA). We performed a Pubmed and Scopus search using the following keywords: “neonate”, “newborn”, “infant”, “*Streptococcus bovis*”, “*Streptococcus gallolyticus*”. An additional manual electronic search was carried out to identify reports not found in our initial search. We used predetermined inclusion and exclusion criteria to find all relevant articles. Reports describing cases of neonatal infection by *S. gallolyticus* were eligible to be included in our review, and there was no restriction on publication year. Reports referring to cases of *S. gallolyticus* infection in any age group other than neonates, reports not including identification of *S. bovis* to the subspecies level, reviews, as well as studies not published in English were excluded from our study. Two authors (KK, MT) independently screened titles and abstracts of the retrieved studies for possible inclusion in the review and then reviewed the articles in depth. Any disagreement was resolved by a third author (RS).

### 3.2. Results

**Figure 1:**
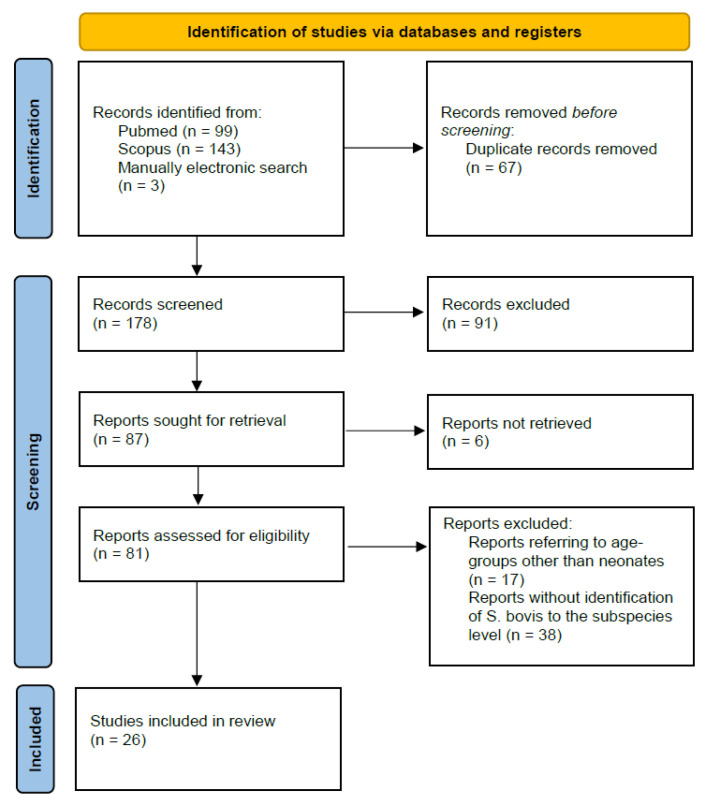
Flowchart of the selection process.

Our systematic search retrieved 245 articles. Duplicates were removed, and the remaining 178 studies were screened for eligibility. Ninety-one records were excluded during the process of title and/or abstract evaluation, and six reports were not accessible in full text to the authors; therefore, eighty-one studies were assessed in full text for possible inclusion in our review. Finally, 26 reports fulfilled the selection criteria and were thus included in our study. The study selection process is depicted in the respective flowchart (Figure 1).

A total of 26 articles reported 66 cases [2,3,4,5,6,7,8,9,10,11,12,13,14,15,16,17,18,19,20,21,22,23,24,25,26,27]. For reasons of completeness, we decided to include in our review three cases of neonatal infection caused by *S. bovis* biotype II, since, according to the bibliography, neonatal infections caused by *S. gallolyticus* species (i.e., *S. bovis* biotype II/2) are much more common than neonatal infections caused by *S. infantarius* (i.e., *S. bovis* biotype II/1). We analyzed these 66 cases and the present case. Characteristics of the cases describing neonatal infection caused by *S. gallolyticus* and *S. bovis* biotype II are summarized in Table 1 and Table 2.

Preterm and term newborns were equally represented among the cases included in this review. Twenty-eight cases (49.5%) referred to preterm neonates (GA 26–36^+6^), and twenty-eight (49.5%) to term neonates. One neonate was post-term, while gestational age was not reported in 10 cases. Regarding the type of delivery, there were 14 cesarean sections (35%), 26 vaginal deliveries (65%), and, in 27 cases, the delivery type was not recorded. Data regarding the length of rupture of membranes were only available in 17 cases; among them, there was only 1 case of prolonged rupture of membranes (preterm premature rupture of the membranes, PPROM). Similarly, details concerning maternal perineal and vaginal cultures were provided in only 18 cases; the majority was GBS negative (12/18, 67%), 3 cases were of unknown GBS status (3/18, 16.5%), 2 cases were GBS positive (2/18, 11%) and 1 case (1/18, 5.5%) was positive for gamma Streptococci that did not belong to Group D. Peripartum antibiotics were used in only 2 cases; one was for premature spontaneous labor at 32 weeks and one for PPROM. However, in most cases (54 out of 67), relevant information was not reported. The time of onset of symptoms was specified in 43 cases. There were 17 cases (39.5%) with early-onset sepsis (data reported in Table 1) and the remaining (60.5%) referred to late-onset sepsis (data reported in Table 2). Among the 67 cases reported, respiratory distress, poor feeding, fever and lethargy were the symptoms most frequently described, followed by seizures, apnea, and symptoms from the gastrointestinal tract. Meningitis was by far the most common complication, being reported in 40 out of 53 cases (75%) (in 14 cases, complications were not listed). Bacteremia complicated with meningitis was reported in 47% (8/17 cases) of early-onset sepsis and in 61.5% (16/26 cases) of late-onset sepsis. Complications from the nervous system also included five cases of ventriculitis (7%), two cases of grade III intraventricular hemorrhage (IVH) (3%), three cases of subdural effusion (4.5%) and one case of hydrocephalus requiring ventriculoperitoneal bypass (1.5%). Regarding the respiratory tract, two cases with persistent pulmonary hypertension were described (3%) (including the present case), one case with pulmonary hemorrhage (1.5%) and one case with pneumonia (1.5%). In almost half of the cases (32 out of 67 cases), *S. gallolyticus* was identified to the subspecies level; in 28 cases (87.5%), *S. gallolyticus* subspecies *pasteurianus* was isolated, and in 4 cases (12.5%) *S. gallolyticus* subspecies *gallolyticus*. Penicillin, or a penicillin-derivative, alone or in combination with another agent (usually cephalosporin or aminoglycoside) was the definitive treatment of choice in 41 out of 56 cases (73%). Among 67 cases, only three neonates (4.5%) died.

## 4. Discussion

This is a case of early-onset neonatal sepsis due to *S. gallolyticus*, with rapid clinical deterioration leading to mechanical ventilation and administration of inotropes, complicated by the development of pulmonary hypertension, with finally benign clinical course and discharge after a 12-day hospitalization.

*S. gallolyticus* belongs to group D Streptococci in the *S. bovis-S. equinus* complex. The taxonomy and nomenclature of group D Streptococci were modified in 2003 [28]. *S. bovis*, based on its ability or inability to ferment mannitol, was designated biotype I (mannitol fermentation-positive) or biotype II (mannitol-fermentation-negative). Biotype II was further divided into biotype II/1 and biotype II/2 depending on further biochemical characteristics. These strains are now known as *S. gallolyticus* subsp. *gallolyticus* (biotype I), *S*. *infantarius* (biotype II/1) and *S. gallolyticus* subsp. *pasteurianus* (biotype II/2). Proper identification is usually accomplished with a combination of various biochemical tests (e.g., API 20 Strep and Rapid ID 32 Strep test system, Vitek 2, matrix-assisted laser desorption ionization-time of flight mass spectrometry-MALDI TOF) with molecular tests (e.g., 16S rRNA and sodA gene sequencing, pulsed-field gel electrophoresis—PFGE) [1]. Based on our literature search, biochemical testing is initially applied to identify bacteria at the species level. However, enzymatic reaction interpretation is not always definite, and this could lead to the misidentification of the isolated strain. Therefore, to minimize the risk of misidentification, various phenotypic tests should be used to verify the results. Moreover, whenever available, genotypic identification of the isolated pathogen with the utilization of molecular tests should be preferred in order to confirm the identity of the isolated strains and to further identify them to the subspecies level. Gene sequencing and PFGE are the methods more widely used to identify the isolated microorganism [29,30] reliably.

In adults, the most common source of infection includes the gastrointestinal and hepatobiliary tracts. *S. gallolyticus* subsp. *gallolyticus* is mainly associated with colonic neoplasia and endocarditis in adults, while *S. infantarius* is linked to non-colonic cancers [31]. Due to the epidemiological correlation between *S. gallolyticus* infection and colon cancer, it is strongly recommended that all adults with *S. gallolyticus* bacteremia are evaluated with colonoscopy [27]. No gastrointestinal disease was known in the mother of the present case; a colonoscopy was scheduled after discharge from the maternity hospital. Recent studies also suggest that contact with infected animals or contaminated food promotes fecal colonization of healthy adults with *S. gallolyticus*, indicating a potential zoonotic origin [32].

The pathophysiology of neonatal infection caused by *S. gallolyticus* species remains unclear. Based on our review of the literature, *S. gallolyticus* subsp. *pasteurianus* is the most common pathogen among *S. gallolyticus* species in neonates. Similarly to GBS, it is believed that colonization of the vagina and vertical transmission to the fetus via an ascending route, especially in case of prolonged rupture of membranes, or transmission to the fetus during parturition, could lead to neonatal colonization and probably infection [33]. In the present case, the exact contagious route was not clarified since no pathogens were isolated in the vaginal swab at 35 weeks of gestation, and there was no prolonged rupture of membranes. Fikar and Levy reported a case of neonatal meningitis caused by *S. bovis*, whose mother had positive vaginal and rectal swabs for the same microorganism [34]. Additionally, Binghuai et al. described a case of intrauterine infection complicated by postpartum bacteremia caused by *S. gallolyticus* subsp. *pasteurianus* in a mother whose infant tested positive for the same infectious agent in ear and throat swabs [35]. This neonate remained asymptomatic, contrary to the cases reported by Sasi et al. [27]. In their study, four out of five neonates born to mothers with intrauterine infection and postpartum bacteremia caused by *S. gallolyticus* subsp. *gallolyticus* had symptoms compatible with sepsis and/ or meningitis. However, no microorganism was isolated from neonatal blood and/ or CSF cultures. Abnormalities of the immune system may further predispose neonates to infections, including those caused by *S. gallolyticus*, as described by Koh et al. and Mettananda et al. [4,19]. Moreover, as stated by Saegeman et al. [2] and Floret et al. [8], nosocomial transmission may play a role in some cases of neonatal infections by *S. gallolyticus* species; this underlines the importance of adherence of medical staff with health protocols and raises awareness of healthcare-associated infections.

A benign clinical course is usually reported for *S. gallolyticus* infections in neonates. Based on our search of the literature, the clinical presentation of *S. gallolyticus* neonatal infection cannot be easily distinguished from GBS infection, with signs and symptoms including sepsis and metabolic acidosis, respiratory distress, apnea and poor overall activity being prominent. Meningitis, which may present with seizures, constitutes a common complication, especially of late-onset sepsis, though it seems to have a favorable outcome. Despite the increased frequency of concomitant bacteremia and meningitis, the neonate of the present case did not have any signs or laboratory evidence of meningitis during its clinical course. Additional complications of the central nervous system described in the literature include ventriculitis, [18,24,26] hydrocephalus [17] and intraventricular hemorrhage (IVH) (two cases with grade III IVH, though one refers to an extremely preterm neonate and hence IVH could be either a complication of the infection or due to underlying prematurity) [13,26]. Complications from the respiratory tract seem to be rare: two cases with pulmonary hypertension have been described (including the present case), [20] one case of pulmonary hemorrhage (referring to a preterm neonate) and one case of pneumonia [27]. Even though only a couple of cases of persistent pulmonary hypertension (PPHN) secondary to *S. gallolyticus* infection have been described thus far, PPHN has been clearly described in the literature as a complication of neonatal sepsis [36]. Release in the bloodstream of bacterial endotoxins, together with inflammatory mediators (including endothelin-1 and thromboxane, which constitute potent vasoconstrictors) and various cytokines, seem to contribute to the pathophysiology of PPHN associated with neonatal sepsis. [37,38] Additionally, although *S. gallolyticus* has been clearly linked with cases of endocarditis in adult patients, to our knowledge, only one case has been reported in the neonatal population; this patient recovered without sequelae [20]. The overall prognosis is encouraging, with three neonatal deaths by *S. gallolyticus* being recorded in the literature out of 67 cases of neonatal infection (including one extremely premature and extremely low birth weight neonate) and 2 cases complicated by severe neurologic sequelae [2,17,25,26]. Postmortem examination findings of the deceased neonates were not described in the literature. Procalcitonin constitutes a promising diagnostic marker of neonatal sepsis, being able to discriminate cases of infection versus non-infectious inflammatory disorders; it could be thus speculated that immunohistochemical techniques, with anti-procalcitonin antibodies, among others, could contribute to the postmortem diagnosis of neonatal sepsis, in cases of diagnostic ambiguity [39,40].

*S. gallolyticus*, in the majority of cases, is sensitive to penicillin and to penicillin derivatives, ref. [5,22] and this was also the case for the neonatal infection caused by *S. gallolyticus* hereby presented. However, we should mention two reports by Khan et al. [7] and Klatte et al. [9] referring to two cases of neonatal meningitis caused by *S. pasteurianus* relatively resistant to penicillin. Therefore, de-escalation of antibacterial therapy after accurate identification of the causative agent and antimicrobial susceptibility testing is legitimate to minimize the risk of multi-drug resistant pathogens. According to our search of the literature, the duration of therapy is variable depending on the clinical course, ranging from a minimum of 5 days [27] to a maximum of approximately 50 days (one case with liver abscess formation [23] and one case with late central nervous system complications) [14].

There are some limitations in our systematic review. In many cases, documentation regarding perinatal history was incomplete, including type of delivery, duration of rupture of membranes, maternal perineal and vaginal swabs and use of prophylactic peripartum antibiotics; consequently, the impact of these perinatal parameters on the pathogenesis of neonatal infection by *S. gallolyticus* cannot be precisely estimated. Additionally, there may be cases of infection by *S. gallolyticus* that have not been captured. Specifically, there are cases of neonatal infection by *S. bovis* in the literature that have not been identified at the subspecies level or cases that may have been misclassified as other Streptococcus species, mainly before the currently available biochemical and molecular techniques. Therefore, the true prevalence of *S. gallolyticus* infection and its subspecies remains unknown.

## 5. Conclusions

In conclusion, the occurrence of *S. gallolyticus* infection in the neonate of the present case report indicates that *S. gallolyticus* may be considered a novel and emerging pathogen in the field of neonatal sepsis. Increased clinical suspicion may be warranted in sepsis cases resembling GBS infections, even in the presence of negative GBS vaginal swab cultures. Precise identification of the pathogens is necessary to detect possible epidemiological changes. More studies are needed to clarify the epidemiology, pathogenesis and possible sequelae of neonatal infections caused by *S. gallolyticus* species.

## Figures and Tables

**Table 1:** Characteristics of cases of early-onset sepsis caused by *S. gallolyticus* or *S. bovis* biotype II.

First Author, Publication Year	GA	Birth Weight (Grams)	Mode of Delivery	Length of Rupture of Membranes	Maternal Vaginal Cultures	Maternal Peripartum Antibiotics	Symptoms Onset Time (Days)	Clinical Signs and Symptoms	Site of Isolation	Diagnosis	Microorganism	Complications	Definitive Antibiotic Treatment	Patient Outcome
Gavin, 2003 [5]	Term	3.925	Vaginal delivery	<18 h	GBS (-)	N/A	3	Fever, irritability, poor oral intake, decreased urine output	Blood, CSF	Biochemical tests (RapID STR, Vitek 2, API 20 Strep, CFA profile)	*S. bovis* biotype II/2	Meningitis, Seizure-like episodes	Penicillin G for 14 days	Full recovery
Khan, 2009 [7]	N/A	N/A	N/A	N/A	N/A	N/A	3	Apnoeic episodes, lethargy	Blood, CSF	Biochemical tests (API 20 Strep kit)	*S. gallolyticus* subsp. *pasteurianus*	Meningitis	Penicillin and gentamicin for 14 days	Recovery
Klatte, 2012 [9]	Term	N/A	Vaginal delivery	3 h	N/A	N/A	2	Poor feeding, lethargy, seizure activity	Blood, CSF	Βiochemical tests (Vitek 2), molecular tests (16S rRNA sequencing)	*S. gallolyticus* subsp. *pasteurianus*	Meningitis	Ampicillin for 16 days, cefotaxime for 6 days	Recovery
Thatrimontrichai, 2012 [11]	39 weeks	3.188	Vaginal delivery	<18 h	Gamma Streptococci not in group D (after onset of neonatal symptoms)	N/A	2	Fever, lethargy, poor feeding, slightly bulging anterior fontanel	CSF, maternal urine	N/A	*S. gallolyticus* subsp. *pasteurianus*	Meningitis, right IVH grade I	Cefotaxime for 14 days and gentamicin for 5 days	Recovery
Beneteau, 2015 [16]	10 preterm and 5 term neonates *	2.160 (1.860–3.430) for neonates with symptoms onset <4 days, 2.240 (1.480–3.570) for neonates with symptoms onset >4 and ≤28 days	N/A	N/A	N/A	N/A	3 neonates ≤ 4 days, 12 neonates >4 and ≤28 days	Fever, hypothermia, digestive signs, respiratory signs, irritability, neurologic signs, sepsis	Blood and/or CSF	N/A	*S. gallolyticus pasteurianus* in 8 cases and *S. gallolyticus gallolyticus* in 2 cases **	Meningitis	Amoxicillin and/or 3rd generation cephalosporin and aminoglycocide for 10–25 days	All neonates survived
Nguyen, 2019 [20]	39^+1^ weeks	4.195	Vaginal delivery	12 h	GBS (-)	N/A	1	Respiratory distress	Blood	N/A	*S. gallolyticus* subsp. *pasteurianus*	Meningitis, endocarditis	Cefepime for 28 days and gentamicin for 14 days	Recovery without sequelae
	40^+1^ weeks	3.250	Vaginal delivery	4 h	GBS unknown	No	1	Poor respiratory effort, irritability	Blood	N/A	*S. gallolyticus* subsp. *pasteurianus*	Septic shock, PPHN	Cefepime and clindamycin for 14 days	Recovery without sequelae
Sim, 2021 [21]	39 weeks	3.374	Vaginal delivery	<18 h	GBS (-)	No	1	Respiratory distress	Blood, CSF	Βiochemical tests (MALDI-TOF)	*S. gallolyticus*	Meningitis	Vancomycin	Recovery without sequelae
Chen, 2021 [22]	35^+1^ weeks	N/A	Cesarean section	N/A	N/A	N/A	3	Apnea, desaturation	Blood	Βiochemical tests (MALDI-TOF), molecular tests (16S rRNA and sodA gene sequencing, PCR-RFLP assays of groESL gene)	*S. gallolyticus* subsp. *pasteurianus*	None	Ampicillin and cefotaxime for 14 days	Recovery without sequelae
	37^+3^ weeks	N/A	Cesarean section	N/A	GBS (+)	No	2	Tachypnea, desaturation, poor activity, fever	Blood, CSF	Βiochemical tests (MALDI-TOF), molecular tests (16S rRNA and sodA gene sequencing, PCR-RFLP assays of groESL gene)	*S. gallolyticus* subsp. *pasteurianus*	Meningitis	Ampicillin and cefotaxime for 14 days	Recovery without sequelae
Geetha, 2021 [23]	36^+6^ weeks	3.776	Vaginal delivery	<18 h	GBS (-)	No	1	Respiratory distress	Blood	Biochemical tests (MALDI-TOF, Vitek)	*S. gallolyticus* subsp. *pasteurianus*	Liver abscess	Cefotaxime for 5 weeks, co-amoxiclav for 3 weeks	Recovery without sequelae
Srour, 2022 [24]	36^+4^ weeks	3.720	Vaginal delivery	<18 h	GBS (-)	N/A	1	Intermittent cyanosis, hypothermia, tachypnea	Blood, CSF	N/A	*S. gallolyticus*	Meningitis, ventriculitis, seizure-like activity	Penicillin G for 21 days	Recovery without sequelae
Williams, 2022 [25]	26 weeks	950	Vaginal delivery	PPROM 12 days	GBS (-)	IV ampicillin for 2 days and pos amoxicillin for 5 days and a single dose of azithromycin	1	Poor respiratory effort, metabolic and respiratory acidosis	Blood	Βiochemical tests (MALDI-TOF)	*S. gallolyticus*	Septic shock	Ampicillin and gentamicin (empirical treatment)	Deceased neonate
Orbea, 2022 [26]	N/A (9 neonates) ***	N/A	N/A	N/A	N/A	N/A	Median age 24 days (1–74 days)	Fever, irritability, difficulty feeding, lethargy, respiratory distress, apnea, seizure-like activity, emesis, diarrhea	Blood and/or CSF	Biochemical tests (MALDI-TOF, Vitek)	*S. gallolyticus*, *S. gallolyticus* subsp. *pasteurianus* ****	Meningitis (11 infants), bilateral grade III IVH (1neonate), ventriculitis and subdural collection and/or subarachnoid debris (3 neonates) *****	Antibiotic therapy for a median of 14 days (9–28 days)	One deceased neonate, one neonate with neurologic complications
Sasi, 2022 [27]	37 weeks	3.125	Vaginal delivery	N/A	N/A	N/A	1	Tachycardia, tachypnea, poor sucking	Maternal blood cultures, placental tissue culture, no growth in neonatal blood culture	Biochemical tests (MALDI-TOF)	*S. gallolyticus* subsp. *gallolyticus*	None	Ampicillin and amikacin for 5 days	Recovery without sequelae
	38 weeks	2.670	Vaginal delivery	3 h	N/A	N/A	1	Tachycardia, tachypnea	Maternal blood cultures, no growth in neonatal blood culture	Biochemical tests (MALDI-TOF)	*S. gallolyticus* subsp. *gallolyticus*	None	Ampicillin and amikacin for 7 days	Recovery without sequelae
	39 weeks	3.620	Cesarean section	N/A	N/A	N/A	1	Respiratory distress	Maternal blood and urine cultures, no growth in neonatal blood culture	Biochemical tests (MALDI-TOF)	*S. gallolyticus* subsp. *gallolyticus*	Meningitis	Ampicillin and amikacin for 10 days	Recovery without sequelae
	41 weeks	4.170	Cesarean section	N/A	N/A	N/A	1	Fever, tachycardia, respiratory distress	Maternal blood cultures, placental tissue culture, no growth in neonatal blood culture	Biochemical tests (MALDI-TOF)	*S. gallolyticus* subsp. *gallolyticus*	Pneumonia	Ampicillin and amikacin for 5 days	Recovery without sequelae
This case	38^+1^ weeks	3.250	Cesarean section	10 h	GBS (-)	No	1	Respiratory distress, poor feeding, fever	Blood	Biochemical tests (Vitek 2)	*S. gallolyticus*	PPHN	Ampicillin and cefotaxime for 10 days	Recovery

Abbreviations: CFA, cellular fatty acid; CSF, cerebrospinal fluid; GBS, group B streptococci; IV, intravenous; IVH, intraventricular hemorrhage; MALDI-TOF, matrix-assisted laser desorption ionization-time of flight mass spectrometry; N/A, not available; PFGE, pulsed-field gel electrophoresis; PCR, polymerase chain reaction; PPHN, persistent pulmonary hypertension of the newborn; RFLP, restriction fragment length polymorphism. * This study included 23 infants, 15 of whom were neonates. ** Subspecies of *S. bovis* were identified in 10 cases. Not specified if the data of *S. gallolyticus* identification refers to neonates or to infants. *** This study included 15 infants, 9 of whom were neonates. **** *S. bovis* was not classified into subspecies in one case. Not specified if the data of *S. gallolyticus* identification refers to neonates or to infants. ***** Not specified if cases of meningitis refer to neonates or to infants.

**Table 2:** Characteristics of cases of late-onset sepsis caused by *S. gallolyticus* or *S. bovis* biotype II.

First Author, Publication Year	GA	Birth Weight (Grams)	Mode of Delivery	Length of Rupture of Membranes	Maternal Vaginal Cultures	Maternal Peripartum Antibiotics	Symptoms Onset Time (Days)	Clinical Signs and Symptoms	Site of Isolation	Diagnosis	Microorganism	Complications	Definitive Antibiotic Treatment	Patient Outcome
Cheung, 2000 [3]	32 weeks	2.340	Vaginal delivery	N/A	N/A	Prophylactic clindamycin	28	Lethargy, possible seizure activity, apnea	Blood, CSF	Biochemical tests (API 20 strep, cellular fatty acid profile)	*S. bovis* biotype II	Meningitis	Ampicillin and gentamicin changed to penicillin for 18 days	Recovery without sequelae
Koh, 2002 [4]	34 weeks	1.675	Cesarean section	N/A	N/A	N/A	19	Lethargy, apnea	Blood, CSF	Biochemical tests (API 20 strep test)	*S. bovis* biotype II	Meningitis	Penicillin G for 14 days	Recovery without sequelae
Onoyama, 2009 [6]	Term	3.192	Vaginal delivery	<18 h	GBS (-)	N/A	4	Fever, poor activity	Blood, CSF	Biochemical tests (API 20 Strep test), molecular tests (16S rRNA and sodA gene sequencing)	*S. gallolyticus* subsp. *pasteurianus*	Meningitis	Cefotaxime for 14 days	Recovery without sequelae
Floret, 2010 [8]	5 preterm	N/A	3 vaginal deliveries, 2 cesarean sections	N/A	N/A	N/A	Median age 18 days (13–56)	Abdominal distention, diarrhea, signs of umbilical inflammation	Blood	Molecular tests (sodA gene sequencing, 16S rRNA gene sequencing, PFGE)	*S. gallolyticus* subsp. *pasteurianus*	N/A	Cefotaxime for 10 days	All neonates survived
Klatte, 2012 [9]	Term	N/A	Vaginal delivery	N/A	N/A	N/A	12	Congestion, increased work of breathing, lethargy	CSF	Βiochemical tests (Vitek 2), molecular tests (16S rRNA sequencing)	*S. gallolyticus* subsp. *pasteurianus*	Meningitis	Cefotaxime for 16 days	Recovery
	Term	N/A	Vaginal delivery	N/A	N/A	N/A	4	Fever, seizure-like activity	Blood, CSF	Βiochemical tests (Vitek 2), molecular tests (16S rRNA sequencing)	*S. gallolyticus* subsp. *pasteurianus*	Meningitis	Ampicillin for 14 days	Recovery
	Post-term	N/A	Vaginal delivery	13 h	N/A	N/A	4	Fever, rhinorrhea, fussiness	Blood, CSF	Βiochemical tests (Vitek 2), molecular tests (16S rRNA sequencing)	*S. gallolyticus* subsp. *pasteurianus*	Meningitis	Cefotaxime for 14 days	Recovery
Nagamatsu, 2012 [10]	Term	3.092	Vaginal delivery	N/A	N/A	N/A	8	Superior tonic eye deviation, fever, irritability, decreased oral intake	CSF	Βiochemical tests (API Rapid ID 32 Strep, VITEK 2), molecular tests (16S rRNA gene sequencing)	*S. gallolyticus* subsp. *pasteurianus*	Meningitis, seizures	Ampicillin for 20 days and panipenem/ betamipron for 14 days	Recovery without sequelae
Tarakci, 2014 [12]	30 weeks	1.300	Cesarean section	N/A	N/A	N/A	37	Apnea, lethargy, cyanosis, superficial respiration	Blood	N/A	*S. gallolyticus* subsp. *pasteurianus*	None	Meropenem for 14 days	Recovery
Hede, 2015 [13]	32 weeks	1.474	Cesarean section	N/A	GBS unknown	No	24	Intermittent tachypnea	Blood	Βiochemical tests (Vitek 2), molecular tests (16S rRNA sequencing, PFGE)	*S. gallolyticus* subsp. *pasteurianus*	None	Ampicillin for 10 days	Survived
	32 weeks	2.120	Cesarean section	N/A	GBS unknown	No	24	Pale color, loose stools, respiratory distress, hypoxemia, hypothermia	Blood, CSF	Βiochemical tests (Vitek 2), molecular tests (16S rRNA sequencing, PFGE)	*S. gallolyticus* subsp. *pasteurianus*	Meningitis, acute respiratory failure, septic shock, seizures, grade III IVH	Ampicillin for 14 days	Survived
Park, 2015 [14]	38^+4^ weeks	3.600	Vaginal delivery	N/A	N/A	N/A	27	Fever, lethargy, moaning sounds	Blood, CSF, urine	Μolecular tests (16S rRNA gene sequencing)	*S. gallolyticus* subsp. *pasteurianus*	Meningitis, bilateral reduction in visual evoked potentials, subdural effusion and seizures	Ampicillin and cefotaxime for 21 days, ampicillin and cefotaxime for 31 days due to CNS complications	Discharge, improvement of visual evoked potential in follow-up
Kennedy, 2015 [15]	37 weeks	3.050	Vaginal delivery	6 h	GBS (-)	N/A	4	Lethargy, irritability, decreased urine output, poor feeding, fever	Blood, CSF	Βiochemical tests (RapID STR), molecular tests (16S rRNA gene sequencing)	*S. gallolyticus* subsp. *pasteurianus*	Meningitis	Ampicillin for 14 days	Recovery without sequelae
Beneteau, 2015 [16]	10 preterm and 5 term neonates *	2.160 (1.860–3.430) for neonates with symptoms onset <4 days, 2.240 (1.480–3.570) for neonates with symptoms onset >4 and ≤28 days	N/A	N/A	N/A	N/A	3 neonates ≤ 4 days, 12 neonates >4 and ≤28 days	Fever, hypothermia, digestive signs, respiratory signs, irritability, neurologic signs, sepsis	Blood and/or CSF	N/A	*S. gallolyticus pasteurianus* in 8 cases and *S. gallolyticus gallolyticus* in 2 cases **	Meningitis	Amoxicillin and/or 3rd generation cephalosporin and aminoglycocide for 10–25 days	All neonates survived
Parain, 2016 [17]	Term	N/A	N/A	N/A	N/A	N/A	28	Altered consciousness, hypertonic limbs, bulging fontanel	CSF	N/A	*S. gallolyticus*	Meningitis, hydrocephalus with tetraventricular dilatation requiring external ventricular drainage, recurrence of hydrocephalus requiring ventriculoperitoneal bypass	Cefotaxime for 17 days and amikacin for 2 days	Recovery with neurologic complications (neuromotor delay with poor spontaneous motor mobilization and hypertonia of the limbs at 9 months)
Saegeman, 2016 [2]	30 weeks	N/A	Vaginal delivery	N/A	N/A	N/A	7	Hemodynamic instability	Blood	Βiochemical tests (MALDI-TOF mass spectrometry), molecular tests (PFGE, 16S rRNA gene sequencing)	*S. gallolyticus* subsp. *pasteurianus*	Meningitis	N/A	Recovery
	32 weeks	N/A	N/A	N/A	N/A	N/A	34	Septic shock, respiratory failure, pulmonary hemorrhage	Blood	Βiochemical tests (MALDI-TOF mass spectrometry), molecular tests (PFGE, 16S rRNA gene sequencing)	*S. gallolyticus* subsp. *pasteurianus*	Septic shock	N/A	Deceased neonate
Yamamura, 2018 [18]	Term	3.680	Vaginal delivery	N/A	N/A	N/A	27	Fever, lethargy, irritability	Blood, CSF	Βiochemical tests (API 20 Strep test), molecular tests (16s rRNA gene sequencing)	*S. gallolyticus* subsp. *pasteurianus*	Meningitis, ventriculitis	Ampicillin for 21 days	Recovery without sequelae
Mettananda, 2018 [19]	34 weeks	1.680	Cesarean section	N/A	N/A	N/A	25	Fever, poor sucking, reduced activity	Blood	Βiochemical tests	*S. bovis* biotype II	Meningitis	Penicillin G and cefotaxime for 21 days	Recovery without sequelae
Sim, 2021 [21]	39 weeks	3.268	Vaginal delivery	<18 h	GBS (-)	No	4	Fever, respiratory distress	Blood, CSF	Βiochemical tests (MALDI-TOF)	*S. gallolyticus*	Meningitis	Ampicillin	Recovery without sequelae
	39 weeks	3.194	Vaginal delivery	<18 h	GBS (-)	No	23	Fever, lethargy	Blood	Βiochemical tests (MALDI-TOF)	*S. gallolyticus*	None	Ampicillin	Recovery without sequelae
	34 weeks	1.812	Cesarean section	<18 h	GBS (-)	No	15	Fever, poor feeding	Blood	Βiochemical tests (MALDI-TOF)	*S. gallolyticus*	None	Clindamycin	Recovery without sequelae
Chen, 2021 [22]	37^+3^ weeks	N/A	Cesarean section	N/A	GBS (+)	No	5	Fever, tachypnea	CSF	Βiochemical tests (MALDI-TOF), molecular tests (16S rRNA and sodA gene sequencing, PCR-RFLP assays of groESL gene)	*S. gallolyticus* subsp. *pasteurianus*	Meningitis	Ampicillin and cefotaxime for 14 days	Recovery without sequelae
Orbea, 2022 [26]	N/A (9 neonates) ***	N/A	N/A	N/A	N/A	N/A	Median age 24 days (1–74 days)	Fever, irritability, difficulty feeding, lethargy, respiratory distress, apnea, seizure-like activity, emesis, diarrhea	Blood and/or CSF	Biochemical tests (MALDI-TOF, Vitek)	*S. gallolyticus*, *S. gallolyticus* subsp. *pasteurianus* ****	Meningitis (11 infants), bilateral grade III IVH (1neonate), ventriculitis and subdural collection and/or subarachnoid debris (3 neonates) *****	Antibiotic therapy for a median of 14 days (9–28 days)	One deceased neonate, one neonate with neurologic complications

Abbreviations: CSF, cerebrospinal fluid; GBS, group B streptococci; IV, intravenous; IVH, intraventricular hemorrhage; MALDI-TOF, matrix-assisted laser desorption ionization-time of flight mass spectrometry; N/A, not available; PFGE, pulsed-field gel electrophoresis; PCR, polymerase chain reaction; RFLP, restriction fragment length polymorphism. * This study included 23 infants, 15 of whom were neonates. ** Subspecies of *S. bovis* were identified in 10 cases. Not specified if the data of *S. gallolyticus* identification refers to neonates or to infants. *** This study included 15 infants, 9 of whom were neonates. **** *S. bovis* was not classified into subspecies in one case. Not specified if the data of *S. gallolyticus* identification refers to neonates or to infants. ***** Not specified if cases of meningitis refer to neonates or to infants.

## Data Availability

Not applicable.
